# Analytic validation and real-time clinical application of an amplicon-based targeted gene panel for advanced cancer

**DOI:** 10.18632/oncotarget.20616

**Published:** 2017-09-01

**Authors:** Michele R. Wing, Julie W. Reeser, Amy M. Smith, Matthew Reeder, Dorrelyn Martin, Benjamin M. Jewell, Jharna Datta, Jharna Miya, J. Paul Monk, Amir Mortazavi, Gregory A. Otterson, Richard M. Goldberg, Jeffrey B. VanDeusen, Sharon Cole, Kristin Dittmar, Sunny Jaiswal, Matthew Kinzie, Suraj Waikhom, Aharon G. Freud, Xiao-Ping Zhou, Wei Chen, Darshna Bhatt, Sameek Roychowdhury

**Affiliations:** ^1^ Comprehensive Cancer Center, The Ohio State University, Columbus, OH, USA; ^2^ Division of Medical Oncology, Department of Internal Medicine, The Ohio State University, Columbus, OH, USA; ^3^ Adena Cancer Center, Chillicothe, OH, USA; ^4^ Orion Cancer Care, Findlay, OH, USA; ^5^ Department of Radiology, The Ohio State University, Columbus, OH, USA; ^6^ Department of Pathology, The Ohio State University, Columbus, OH, USA; ^7^ University Pathologists, LLC, Department of Pathology, Roger Williams Medical Center, Providence, RI, USA

**Keywords:** genomics, precision medicine, oncology, molecular diagnostics, sequencing

## Abstract

Multiplex somatic testing has emerged as a strategy to test patients with advanced cancer. We demonstrate our analytic validation approach for a gene hotspot panel and real-time prospective clinical application for any cancer type. The TruSight Tumor 26 assay amplifies 85 somatic hotspot regions across 26 genes. Using cell line and tumor mixes, we observed that 100% of the 14,715 targeted bases had at least 1000x raw coverage. We determined the sensitivity (100%, 95% CI: 96-100%), positive predictive value (100%, 95% CI: 96-100%), reproducibility (100% concordance), and limit of detection (3% variant allele frequency at 1000x read depth) of this assay to detect single nucleotide variants and small insertions and deletions. Next, we applied the assay prospectively in a clinical tumor sequencing study to evaluate 174 patients with metastatic or advanced cancer, including frozen tumors, formalin-fixed tumors, and enriched peripheral blood mononuclear cells in hematologic cancers. We reported one or more somatic mutations in 89 (53%) of the sequenced tumors (167 passing quality filters). Forty-three of these patients (26%) had mutations that would enable eligibility for targeted therapies. This study demonstrates the validity and feasibility of applying TruSight Tumor 26 for pan-cancer testing using multiple specimen types.

## INTRODUCTION

Real-time genomic testing for patients with cancer is central to identifying actionable somatic alterations that can guide clinical decision-making for novel therapies in clinical trials [1]. A recent retrospective analysis of 570 Phase II clinical trials with over 30,000 patients illustrates that biomarker-driven selection of personalized therapies results in significantly improved objective response rates, progression-free survival, and overall survival compared to non-personalized therapies [2].To enable ongoing drug development in trials that utilize such predictive biomarkers, genomic testing strategies ideally should be cost-effective, have a rapid turnaround time, be applicable to multiple sample types, and meet common laboratory standards for clinical decision-making. To achieve these goals, diagnostic laboratories are implementing multi-gene panels on next generation sequencing platforms. Here, we describe the evaluation and implementation of a multi-gene panel in our Clinical Laboratory Improvements Amendment (CLIA) certified Cancer Genomics Laboratory at The Ohio State University Comprehensive Cancer Center.

We selected the Illumina MiSeq sequencing platform due to its desktop scale, high accuracy, and sufficient throughput [3] and evaluated Illumina's TruSight Tumor 26 (TST) amplicon kit, which targets 85 hotspot regions in 26 genes commonly altered in cancer. To analytically validate the performance of this assay, we utilized three cohorts of positive control samples: cancer cell lines, fresh frozen tumor tissues, and formalin-fixed paraffin-embedded (FFPE) tumor tissues. We created mixes for each of the three specimen types to generate samples with several unique mutations at predicted variant allele frequencies (VAFs) of 3 - 100% to maximize cost-efficiency and reduce the number of samples needed to complete the analytic validation. These sample mixes were used to assess the sensitivity, positive predictive value (PPV), precision, and limit of detection of the TST assay to detect single nucleotide variants (SNVs) and small insertions and deletions (indels).

Subsequently, we applied this analytically validated assay for patients seen in a medical oncology clinic in real time. Prospective patients were consented to a clinical sequencing study that includes permission to use a previous FFPE biopsy or obtain a new research tumor biopsy for genomic testing of both tumor and germline specimens and return of results [4].Using this approach, we evaluated 174 consecutive patients with any type of advanced or metastatic cancer. We assessed the quality of the tumor samples, DNA, amplified libraries, sequencing metrics, and VCF files. Given the volume of testing and results that require interpretation, we utilized a curated database, Cancer Driver Log (CanDL) to support rapid clinical interpretation of somatic results and to flag clinically relevant mutations [5].This study demonstrates the feasibility of obtaining research tumor biopsies in real-time for clinical care and the potential impact of targeted genomic testing on clinical decision-making.

## RESULTS

### Generation of sample mixes for analytic validation

**Figure 1:**
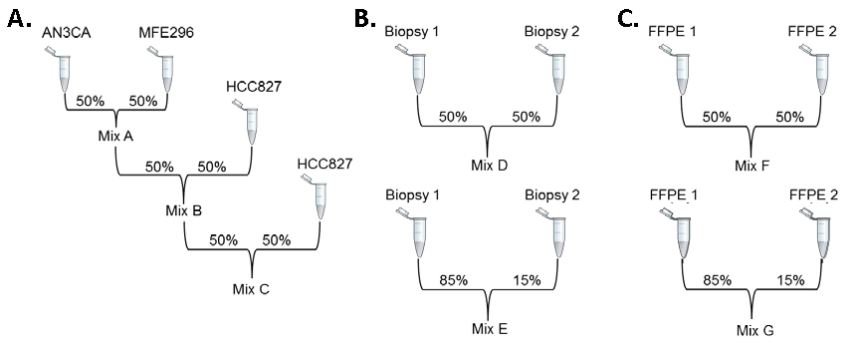
Generation of sample mixes for analytic validation DNA from seven original samples was diluted to generate seven mixes. **A.** AN3CA and MFE-296 cell lines were mixed 1:1 to create Mix A, which was then mixed 1:1 with HCC827 to create Mix B and once again to create Mix C. **B.** Two frozen tumor samples and **C.** two FFPE tumor samples were combined 1:1 and 85:15 to generate mixes D and E (frozen) and F and G (FFPE).

Three extensively sequenced cancer cell lines (AN3CA, MFE-296, and HCC827) were analyzed by TST in our Cancer Genomics Laboratory. All SNVs and indels previously reported in the Cancer Cell Line Encyclopedia (CCLE) or the Catalogue of Somatic Mutations in Cancer (COSMIC) databases that were within the TST targeted regions were detected, and additional variants identified by TST were Sanger sequenced to confirm their presence (data not shown). DNA from these three cell lines was then combined as indicated in Figure 1 to generate three DNA mixes, containing 23 (Mix A), 25 (Mix B), and 26 (Mix C) unique variants at predicted VAFs > 3%. Similarly, to extend the validation to expected sample types, DNA from two frozen tumor biopsies and two FFPE tumor biopsies was analyzed independently, and all variants detected by TST were Sanger sequenced to confirm their presence. These DNA samples were then combined as indicated in Figure 1 to generate DNA mixes containing a total of 28 frozen (14 Mix D and 14 Mix E), and 25 FFPE (15 Mix F and 10 Mix G) mutations at expected VAFs > 3%. The 127 mutations identified in these seven mixes were used as “condition positives” to analytically validate Illumina's TST assay, which amplifies 85 hot spot regions within 26 genes (Supplementary Table 1). The VAFs of mutations detected in the individual cell lines, frozen tumors, and FFPE tumors used for mix generation are listed in Supplementary Table 2.

### NGS data quality and coverage

Libraries were generated from fourteen DNA samples or mixes. Some were prepared multiple times to evaluate assay precision, resulting in a total of 40 independent sample preparations constituting 80 libraries (an A and B library for each). Dual strand libraries help to evaluate PCR or formalin-fixation induced artifacts. Sequencing these libraries on Illumina MiSeq instruments resulted in the following ranges of quality metrics: cluster density = 1241 to 1372, clusters passing filter = 83.6 to 93.8%, and reads > Q30 = 93.8 to 96.1%. Overall yields ranged from 5.01 to 6.03 gigabases (Gb) per run, which represents 1.95 to 3.55 million reads passing filter per sample.

**Figure 2:**
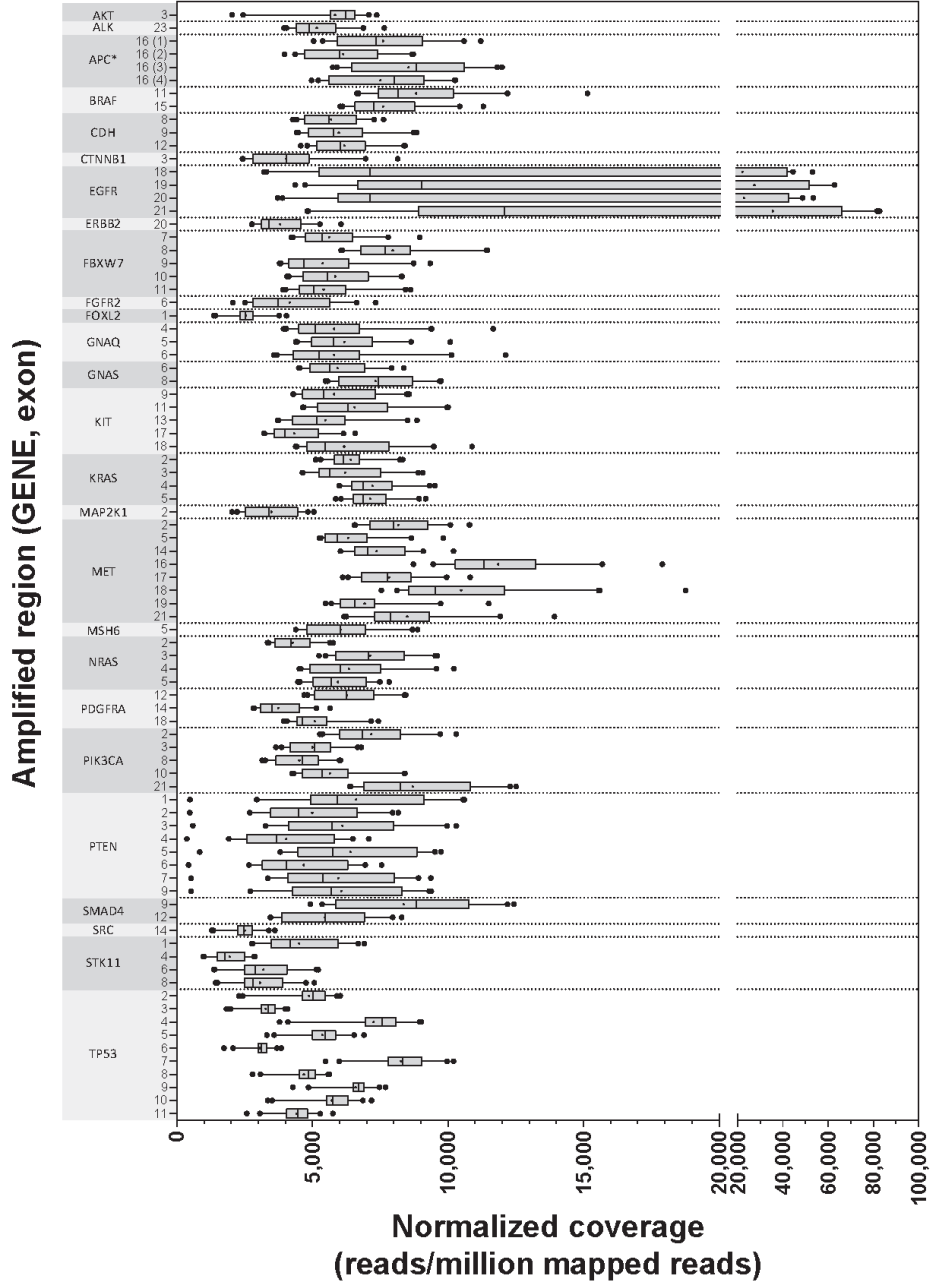
Normalized coverage by amplicon Forty independently prepared libraries were sequenced for analytic validation of TST, and the mean normalized read depths for each of 85 regions are illustrated with the gene and exon covered listed on the Y axis. Box boundaries represent 25th and 75th percentiles, vertical lines within the boxes represent the median, a plus sign (+) within the box represents the mean, and the whiskers represent 5th and 95th percentiles. Outliers beyond 5th and 95th percentiles are illustrated as solid dots. *All exons are covered by one amplified region except for APC exon 16, which has four non-contiguous targeted regions.

The TST assay includes 14,715 targeted bases, and all were routinely covered with at least 1000 reads. Any regions with low coverage were sample specific, and therefore, likely represent true deletions within these samples and not systematic limitations of the assay. Figure 2 illustrates the range of mean normalized read depths (reads/million mapped reads) across all 40 datasets for each of the 85 targeted regions. Similarly, Supplementary Figure 1A shows the range of mean raw read depths across the same 40 runs.

### Sensitivity and positive predictive value

**Figure 3:**
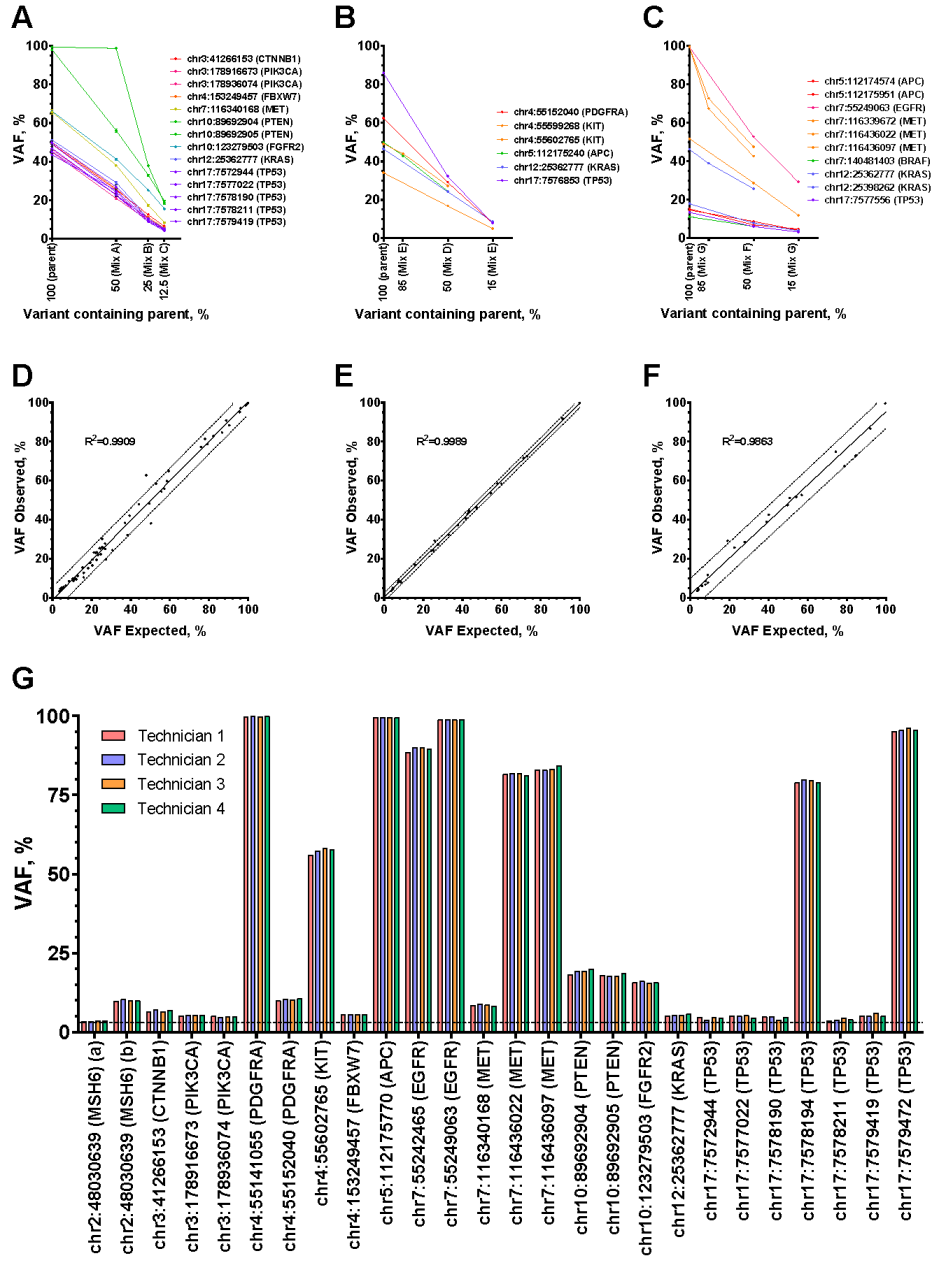
Sensitivity, precision, and limit of detection **A.**-**C.** Variant allele fractions for the mutually exclusive variants were plotted for each sample (parent or mix) for cell lines **A**., frozen tumors **B.**, or FFPE tumors **C.** The x-axis shows percentage of variant containing parents for ANC3A, MFE296, frozen biopsy, and FFPE samples from the Figure 1 dilution series. **D.**-**F.** Calculated expected variant allele fractions were plotted against observed variant allele fractions for cell lines **D.**, frozen tumors **E.**, and FFPE tumors **F.** Linear regressions yielded coefficients of determination (R^2^) of 0.9909, 0.9989, and 0.9863. **G.** Four technicians independently prepared and sequenced Mix C samples using the TST assay. All 26 variants with expected variant allele fractions of > 3% were consistently detected and the variant allele fractions are plotted for each technician. A hashed line at 3% indicates the default limit of detection of the assay.

Using the seven cell line, frozen tumor, and FFPE tumor mixes, TST detected all 127 variants > 3% expected VAF and did not detect any additional variants, resulting in a combined sensitivity of 100% (95% CI: 96-100%) and PPV of 100% (95% CI: 96-100%) (Table 1). In addition to determining variant presence or absence, we examined the VAFs of the mutually exclusive mutations in cell line (14), fresh frozen (6), and FFPE (10) dilution mixes. Each of the three specimen type mixes resulted in anticipated decreases in VAFs, with variants detected as low as 3.17, 5.15, and 3.14% VAF, respectively (Figure 3A-3C). While most variants diluted as expected, we noted that Mix A had one outlier (Figure 3A, *PTEN*), which can be explained by the fact that VariantStudio software (Illumina) annotates deletions at the base position prior to the deletion. In this case, the AN3CA cell line has a deletion in *PTEN* at genomic position chr10:89692905, but this deletion is attributed to chr10:89692904. Since the MFE-296 cell line contains a missense variant at chr10:89692905, when mixed 1:1 with AN3CA the read depth decreases by ~50% (data not shown), but the reported VAF remains the same.

**Table 1:** Accuracy and precision summary

Sensitivity							
**Dilution**	**Sample Type**	**True Positives**	**Technicians**	**MiSeqs**	**# of Runs**	**SNVs detected**	**Sensitivity** **(95% CI)**
	Cell line mixes (A,B,C)	74	4	2	4	74/74	100% (94 - 100%)
	Frozen tumor mixes (D,E)	28	1	1	1	28/28	100% (85 - 100%)
	FFPE tumor mixes (F,G)	25	1	1	1	25/25	100% (83 - 100%)
	All	127	4	2	6	127/127	100% (96-100%)
	**Sample**	**True Positives**	**Technicians**	**MiSeqs**	**# of Runs**	**SNVs detected**	**Sensitivity** **(95% CI)**
**Down-sampling**	Mix C - 28 tiles	26	1	1	1	26/26	100% (84 – 100%)
	Mix C - 3 tile	26	1	1	1	26/26	100% (84 – 100%)
**Positive Predictive Value**							
**Comparison**		**True Positives**	**Technicians**	**MiSeqs**	**# of Runs**	**SNVs detected**	**PPV** **(95% CI)**
All samples		127	4	2	6	127	100% (96-100%)
**Precision**							
**Comparison**		**SNVs**	**Technicians**	**MiSeqs**	**# of Runs**	**SNVs detected**	**Concordance**
Repeatability Intra-tech		26	1	1	1	26/26	100%
Reproducibility Inter-Miseq		26	1	2	2	26/26	100%
Reproducibility Inter-tech		74	4	2	4	74/74	100%

Next, we compared the observed VAFs to expected VAFs after dilution in mixes (Figure 3D-3F). Expected values < 3% were below the TST reporting threshold and were therefore excluded, leaving 74 (cell line), 28 (frozen), and 25 (FFPE) variants. All expected variants were detected, and linear regression of the values for each specimen type revealed coefficients of determination (R^2^) of 0.9909, 0.9989, and 0.9863 for cell lines, frozen tumors, and FFPE tumors respectively.

### Measurement of assay precision

Next, we evaluated the within-run repeatability and between-run reproducibility of TST. Four independent Mix C DNA samples were prepared by one technician at the same time, and the resulting libraries were sequenced on two MiSeq instruments (Table 1 and Supplementary Table 3). Then, to assess inter-technician reproducibility, four technicians independently prepared and sequenced Mix A, B, and C libraries (data shown only for Mix C), and 100% concordance was observed for the 74 mutations > 3% VAF across experiments (Figure 3G, Table 1, and Supplementary Table 4).

### Limit of detection

While TST consistently detected all expected variants > 3% VAF when performed following manufacturer's recommendations, we sought to determine how this sensitivity might be impacted at lower read depths. To investigate this, we computationally down-sampled the data from one MiSeq run of four independently prepared Mix C samples. This was done by manually selecting a decreasing number of tiles to include in the analysis. One entire MiSeq flow cell is divided into 28 tiles, and when using all tiles median coverage of the 26 Mix C variants > 3% VAF was 25,822 (min = 8,417, max = 286,577). We compared this complete dataset to other randomly selected data sets consisting of 14 tiles, 7 tiles, 3 tiles, or 1 tile. Supplementary Tables 5 (VAF) and 6 (read depth) illustrate that all 26 expected variants > 3% VAF were detected in all four replicates when using 14, 7, or 3 tiles with corresponding median coverage across the 26 Mix C variants of 13,109, 6,621, and 2,727, respectively. When using 1 tile, median coverage decreased to 955 (min = 311, max = 10,092), and 19 of 26 variants were still consistently detected.

### Application for real-time clinical tumor sequencing

**Figure 4:**
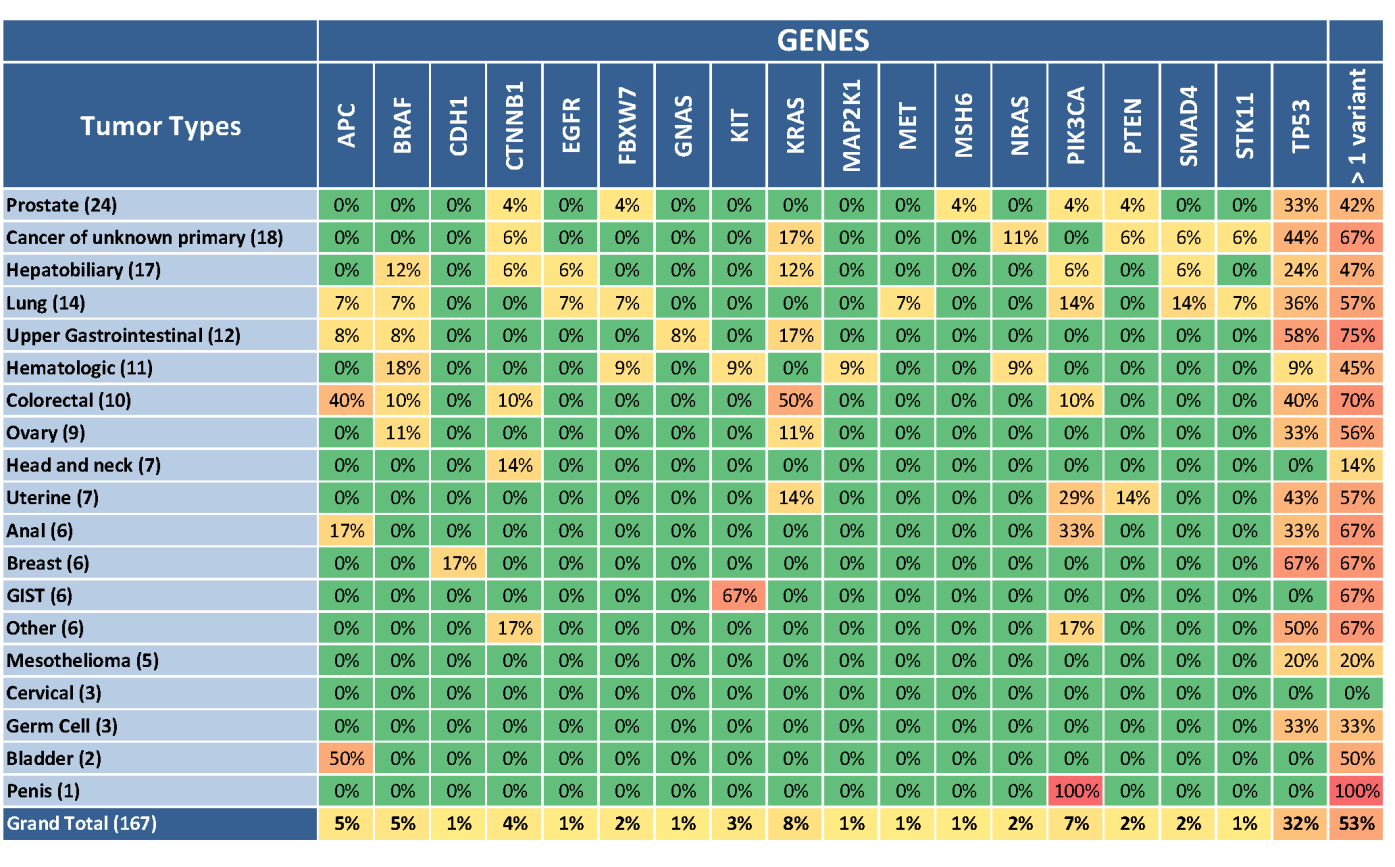
Mutations observed across the patient cohort A total of 167 patients with diverse cancer types were successfully evaluated with the TST assay and reportable variants were detected in 89 (53%). Here, patients are grouped by tumor type, and the number of patient samples of each tumor type successfully processed with TST is shown in parentheses. The percentage of these samples with a somatic alteration is listed for each gene and for each tumor type across all genes (last column). Of the 26 genes targeted by TST, only the 18 genes with detected variants are illustrated.

Following analytical validation of TST, we applied this assay to prospective patient samples in real time in our CLIA-certified laboratory. We examined 174 patients including 69 patients (40%) with archival FFPE specimens, 104 patients (60%) with fresh frozen specimens from a new tumor biopsy, and one patient (0.6%) with PBMC isolated from a peripheral blood specimen. All patients had previously undergone standard of care gene testing appropriate for their respective cancer types, e.g. Sanger sequencing or FISH. DNA was isolated from both normal (peripheral blood or buccal) and tumor samples for each patient, and two patients (eight libraries including an A and B library for each sample) were multiplexed per MiSeq run. Only 7 patient samples failed to pass all quality control metrics, resulting in 167 patients (96%) with reportable results (Supplementary Table 7). Of these, 89 patients (53%) had at least one reportable variant. Figure 4 illustrates a summary of the cancer types and gene variants identified across the 85 amplified regions. As expected, the most commonly mutated genes across all cancer types were *TP53* (33% of tumors) and *KRAS* (9% of tumors). Forty-three patients (26%) had variants detected in their tumors that conferred eligibility to receive targeted therapy (Table 2).

**Table 2:** Patients with potential targeted therapy available

Gene	Number of Patients	Tumor Types
BRAF	8	cholangiocarcinoma, colorectal, duodenum, Hodgkin disease, lung, Langerhans cell histiocytosis, and ovary
EGFR	2	cholangiocarcinoma and lung
FBXW7	1	prostate
KIT	5	chronic myelogenous leukemia and gastrointestinal stromal tumor
KRAS	14	cancer of unknown primary, cholangiocarcinoma, colon, duodenum, ovary, pancreas, rectum, uterus
MAP2K1	1	hairy cell leukemia
MET	1	lung
MSH6	1	prostate
NRAS	3	cancer of unknown primary, Hodgkin disease
PIKC3A	11	anus, cholangiocarcinoma, endometrium, lung, penis, peritoneum, prostate, rectum, uterus
PTEN	2	cancer of unknown primary and endometrium
STK11	2	cancer of unknown primary and lung

## DISCUSSION

We have demonstrated a cost-effective analytic validation of a commercially available amplicon-based cancer gene hotspot panel and successfully applied this assay to prospective patients in real-time using multiple specimen types. The TST assay aims to amplify 85 somatic hotspot regions across 26 genes including 14,715 targeted bases, and in our validation samples, we observed that 100% of targeted bases routinely had at least 1000x coverage. Using cell line and tumor DNA mixes, SNVs (27) and indels (8) were detected with 100% sensitivity and 100% positive predictive value at variant frequencies greater than or equal to 3% and coverage of at least 1,000x. The reproducibility of this assay was also 100% across four technicians and two MiSeq instruments. Not only were SNVs and small insertions and deletions reliably detected down to 3% variant allele fraction, but variant frequencies across runs from different technicians and MiSeq instruments were quite consistent (standard deviations < 1.2%).

This amplicon-based cancer gene panel performed comparably to several published amplicon designs for archival tumor specimens [6–10].The majority of these assays include hotspots for 26-54 genes with average coverages of 500-10,000x. Collectively, these assays appear to be highly accurate and precise and permit the use of multiple specimen types including FFPE samples. Their small target region sizes enable relatively deep coverage even on a desktop sequencer, which allows for reliable detection of variant allele fractions as low as 3-10%. In addition, amplicon DNA input requirements could be as low as 10 ng, which enables the evaluation of small specimens such as fine needle biopsies. Another advantage of commercially available gene panels is that they include established and optimized protocols and bioinformatics workflow support. Furthermore, since gene panels focus on routinely tested oncogenes and tumor suppressor genes, detected somatic variants are more likely to be clinically relevant and less likely to be variants of unknown significance than those from larger panels encompassing whole genes or additional genes with less clear biomarker applications.

Thus far, all previous studies of commercially available amplicon assays have reported on samples from archival cohorts of cancer including solid tumors and hematologic malignancies [6–10]. Therefore, to evaluate the feasibility of prospectively applying an amplicon-based gene panel in clinical practice, patients with advanced or metastatic cancer were consented to real-time clinical tumor sequencing for paired tumor and normal germline testing. Thus far, we have applied this assay prospectively to 174 patients with metastatic or advanced cancer. When applied to patient specimens in real-time, 96% of samples yielded quality libraries and sequencing data. Mutations with VAFs > 3% were identified in tumors with tumor cell fractions ranging from 10 - 90%. The assay was successfully applied to diverse cancer types as well as multiple specimen types including frozen tumors, formalin fixed tumors, and enriched peripheral blood mononuclear cells (PBMCs) in hematologic cancers.

In patient samples, the ranges of mean raw read depth of each targeted region (Supplementary Figure 1B) were more variable than in the validation sample cohort (Supplementary Figure 1A). However, in 334 samples across 85 amplified regions, only 80 of 28,390 total regions had mean raw read depths of less than 1000x, and many of these may represent actual deletions. While this assay was not analytically validated here for the determination of copy number variation, outlier coverage suggested amplification of *EGFR* in HCC827, which has been previously reported [11], and deletion of *PTEN* in “Biopsy 2” used for frozen tumor mixes (not confirmed). Likewise, we detected similar read depth increases in *EGFR* (patients 9 and 59) and *ERBB2* (patients 55 and 111) as well as reduced coverage of *PTEN* in several patient samples (patients 4, 19, 38, 61, 85, 123, and 142) suggesting that this assay may provide evidence for gene amplifications and deletions. Detecting copy number alterations from amplicon-based NGS panels has been previously reported using an Ion Torrent platform [12]. Thus, the TST assay could be further investigated for the potential detection of recurrent copy number alterations involving *ERBB2*, *EGFR*, *PTEN*, *FGFR2*, and *TP53*.

As expected, the most commonly observed somatic mutations in patients occurred in *TP53* (33% of tumors). We also observed several mutations in the MAP kinase signaling pathway (14% of tumors), involving *KRAS*, *NRAS*, *BRAF,* and *MAP2K1* oncogenes across multiple cancer types including hematologic, lung, ovarian, duodenal, biliary, and colorectal (Figure 4, Table 2, Supplementary Table 7). Using the Cancer Driver Log database (www.candl.osu.edu) [5] and previous reports [13–15], these mutations were interpreted as kinase-activating mutations, which provided eligibility for these patients to potentially proceed to a matching therapy. For instance, tumors harboring mutations known to result in constitutive activation of mitogen-activated protein kinase kinase (MEK) [13–15] may benefit from treatment with a MEK inhibitor. Similarly, a patient with neuroendocrine cancer of the bowel was found to have an activating mutation of *BRAF* (p.V600E) and became eligible for a RAF inhibitor clinical trial. For the PI3K pathway, we observed alterations in 7% of tumors involving gain-of-function *PIK3CA* hotspots (Figure 4, Table 2, Supplementary Table 7), or loss-of-function mutations (and likely copy number deletions) of *PTEN*.

Furthermore, the assay proved effective in detecting mechanisms of acquired resistance with secondary mutations involving oncogenes. Of five patients with metastatic gastrointestinal stromal cell tumors who were resistant to multiple tyrosine kinase inhibitors, three had secondary resistance mutations in *KIT* detected by TST that may lead to changes in clinical management with the selection of an appropriately sensitive inhibitor. These cases demonstrate the advantage of pan-cancer testing with multi-gene panels and the real-time impact of molecular testing from tumor biopsies on decision making for progressive disease.

While commercially prepared amplicon assays can be more expensive than in-house designed capture strategies, our down-sampling data suggest that the assay provides significantly more sequencing read depth than is typically necessary, and one might be able to double or perhaps even quadruple the number of libraries multiplexed together onto one MiSeq flow cell, thus reducing the cost per sample by 50-75%.

Overall, we found the TST assay to have a straightforward bench workflow, automated analysis onboard the MiSeq, 100% sensitivity down to 3% variant allele fraction, and excellent reproducibility. Using this assay, we successfully detected 134 reportable somatic mutations within 89 of 167 patient samples, potentially allowing 43 patients to become eligible for additional targeted clinical therapy.

## MATERIALS AND METHODS

### Cell lines

AN3CA (ATCC HTB-111) cells were obtained from the American Type Culture Collection (ATCC, Manassas, VA) and cultured in Eagle's Minimum Essential Medium supplemented with 10% fetal bovine serum (FBS) (Sigma Aldrich, St. Louis, MO). MFE-296 cells (Sigma Aldrich) were cultured in minimal essential media (MEM) (Sigma Aldrich) supplemented with 2 mM L-Glutamine (Sigma Aldrich) and 10% Fetal Bovine Serum (Sigma Aldrich). HCC827 cells (ATCC CRL-2868) were obtained from ATCC and cultured in RPMI-1640 medium (Life Technologies, Carlsbad, CA) supplemented with 10% fetal bovine serum (FBS) (Sigma Aldrich). All three lines were incubated at 37°C and 5% CO2 per manufacturers’ protocols and harvested for DNA extraction when ~ 80% confluent.

### Patient specimens

Patients were consented and enrolled in OSU-13053: Personalized Cancer Medicine Through High-Throughput Sequencing (NCT02090530), which was approved by the Institutional Review Board of the Ohio State University Wexner Medical Center [4]. Peripheral blood was collected from each patient to use as a germline comparison. For patients with archival formalin fixed paraffin embedded (FFPE) tumor blocks from a previous biopsy or resection, these were sectioned (1 × 5 μm section) and mounted onto a glass slide for hematoxylin and eosin (H&E) staining. Viable tumor content was assessed by a board-certified pathologist (A.G.F., X.P.Z.), and specimens with at least 10% tumor tissue were considered for molecular testing. Three 10 μm unstained sections were used for DNA isolation. For patients undergoing research tumor biopsies through interventional radiology, technicians were on site to facilitate immediate collection and freezing of individual samples (generally 6-8) in cassettes containing optimum cutting temperature (OCT) medium (VWR, Radnor, PA). Similar to FFPE blocks, needle biopsies were sectioned (1 × 5 μm section), mounted, and H&E stained to assess for viable tumor content. Generally, the biopsy with the highest tumor content was selected for DNA extraction (using the entire biopsy) and library preparation. For patients with hematologic malignancies predominantly involving blood or bone marrow, a Ficoll gradient was used to separate neutrophils (germline) from peripheral blood mononuclear cells (tumor).

### DNA extraction and QC

DNA was isolated from cell lines and frozen tumors using QiaAMP DNA mini kit (Qiagen, Hilden, Germany), from blood using QiaAMP blood mini kit (Qiagen), and from FFPE tumors using QiaAMP DNA FFPE Tissue kit (Qiagen) per manufacturer's instructions. A Qubit fluorometer (ThermoFisher, Waltham, MA) was used to quantitate DNA. To adjust for variations in DNA quality from various types of specimens, the TST (Illumina, San Diego, CA) protocol uses QPCR and compares the quantitation cycle (Cq) of the sample to the Cq of a control DNA to determine the amount of DNA required as input for the assay instead of recommending a fixed input DNA quantity. Patient samples that failed this QPCR requirement were still processed using the maximum amount of DNA as input because we observed that libraries passing QC were often still generated.

### Preparation of DNA mixes

To assess several mutations across different variant allele fractions, particularly those in the 3-20% range, we generated seven sample mixes. First, we selected three cell lines from an initial set of 20 cell lines processed with TST. HCC827 was selected because it contains a 15 bp *EGFR* deletion accompanied by copy number amplification of the *EGFR* gene [11]. AN3CA and MFE-296 cell lines were selected because they had the maximum number of unique mutations detected by TST that were also absent in HCC827 in order to provide several variants that would be present in the 3-20% range in the final mix. Since all three cell line DNAs were of high quality based on the recommended QPCR assessment, each DNA was diluted to 50 ng/μl. Mix A was generated by mixing an equal volume of AN3CA DNA and MFE-296 DNA. Mix B was subsequently made by mixing an equal volume of Mix A with HCC827 DNA. Finally, Mix C was prepared by mixing an equal volume of Mix B with HCC827 DNA. Five μl (250 ng) DNA was used as input for the TST assay.

To assess limit of detection in patient samples, similar mixes were made using DNA from either frozen tumor biopsies or FFPE samples. Specifically, two frozen and two FFPE samples containing somatic variants detected with TST were selected for mix preparation. To conserve sample and maximize range of variant allele fractions, two mixes were prepared for each of these sample types containing either 50:50 (Mixes D and F) or 85:15 (Mixes E and G) of sample 1 DNA and sample 2 DNA respectively.

### Library preparation

The TST kit (Illumina) was used to prepare next generation sequencing (NGS) libraries containing 178 amplicons targeting 85 hotspot regions in 26 genes. Manufacturer's recommendations were followed to generate an A and B library for each DNA sample to ensure that a variant is identified by both libraries and is not the result of an artifact in one library. Briefly, input DNA was independently hybridized to A and B amplicon primers in separate tubes, unbound oligos were removed, and bound oligos were extended and ligated to generate a PCR template containing universal binding sites for the index primers. Targeted regions were then simultaneously PCR amplified using index-containing primers, which also contain the P7 and P5 regions required for sequencing on the MiSeq. After a PCR cleanup step, the quality of amplified samples was assessed using a TapeStation (Agilent, Santa Clara, CA) to determine library size and a Qubit to determine library concentration.

### Sequencing

Eight libraries (A and B from four samples) were multiplexed and sequenced using one of two MiSeq instruments (Illumina) and one v2 300-cycle sequencing kit (Illumina). Manufacturer's recommendations were followed to load 600 μl of 10 pM TST library mix (6 fmol) containing 0.2 pM PhiX per run.

### Sequence analysis

FASTQ data for each library were generated automatically on MiSeq instruments by MiSeq Reporter software (Illumina) using the index sequences listed in the corresponding “Sample Sheet” for each run. Analysis of the FASTQ data also proceeded automatically on the MiSeq to generate BAM and VCF files for each library and for the combined A and B libraries for each sample. The MiSeq Reporter software generates a genome.vcf file that contains information about the coverage, quality, and variant allele fraction of every targeted base and another VCF file that contains details about only the variants identified. The genomic variants identified were subsequently annotated by importing these VCF files into VariantStudio 2.2.1 software (Illumina). Annotated variants were then exported into Excel using the “All Transcripts for Variant” option to identify tumor specific variants and compare variants across all samples. Variants were eliminated if they did not pass all of Illumina's preset filters for probe bias, strand bias, and read depth.

### Coverage assessment

The automatically generated genome.vcf files were utilized to identify variant allele fraction and read depth in all positions whether or not a variant was detected. Since the total number of mapped reads varied from one run to another, coverage (read depth) was normalized to compare across samples. Normalized coverage was calculated by multiplying the total run yield Gb by the fraction of each indexed library to generate the number of reads supporting each of the eight libraries in that run. The reads for library A and B for each sample were then combined to generate the total mapped reads supporting each sample. Then, the read depth at each targeted base position (from the genome.vcf file) was multiplied by 1E^^6^ and divided by the total number of mapped reads for that sample to generate the number of reads per million mapped reads for each targeted base.

To investigate the range of coverage for each targeted region across the validation set of 40 samples, the mean read depth (either normalized or raw) was calculated for each sample for each of the 85 targeted regions. A box and whisker plot was then created to visualize the interquartile ranges, 5%, 95%, median, and mean of read depths for each region. A similar plot was generated for raw read depths for the 334 patient samples evaluated (167 tumor and normal pairs).

### Sensitivity and positive predictive value assessment

Sensitivity and positive predictive value were assessed using the cell line mixes (A, B, and C), frozen mixes (D and E), FFPE mixes (F and G), and the seven parental samples used to create these mixes. To simplify visualization of variant allele fractions across dilutions, only mutually exclusive variants in all mixes were plotted. Variants were investigated independently for cell lines, frozen tumors, and FFPE tumors.

Expected variant allele fractions were calculated for all variants (including those not mutually exclusive) in all mixes using the normalized coverage (NC) and variant allele fraction (VAF) of its two parent samples:

( (VAF1 x NC1 × 0.5) + (VAF2 x NC2 × 0.5) ) / ( (NC1 × 0.5) + (NC2 × 0.5) ) or

( (VAF1 x NC1 × 0.85) + (VAF2 x NC2 × 0.15) ) / ( (NC1 × 0.85) + (NC2 × 0.15) ). Normalized coverage was used for this assessment in order to account for possible copy number amplifications and deletions that would impact the expected variant allele fraction. Calculated expected variant allele fractions were plotted against the actual variant allele fractions detected in each mix (for cell lines, frozen, and FFPE samples) and a linear regression was fit to each set of data to generate a coefficient of determination, R^2^.

### Assessment of assay precision

One technician processed four separate 250 ng samples of Mix C DNA in one run to assess intra-technician repeatability. The same eight libraries were then processed on a second MiSeq instrument to assess inter-MiSeq reproducibility. Finally, four technicians processed the same Mix A, Mix B, and Mix C samples to assess inter-technician reproducibility. Concordance of variants and their variant allele fractions were compared between runs.

### Limit of detection

To address the level of coverage required for variant detection at a range of variant allele fractions, we manually and randomly down-sampled the raw reads from one run of four intra-technician replicates of Mix C. The configure.xml file in the MiSeq output folder lists 28 tiles; thus, each tile corresponds to 1/28th of the total reads for the run (consisting of eight libraries). By selectively removing individual tiles and re-queuing the sample with MiSeq Reporter software, we iteratively investigated the detection of variants using 14 (50%), 7 (25%), 3 (10.7%), or 1 (3.6%) tile(s) for each of the four Mix C replicates.

### Data availability

All FASTQ files will be made available upon request.

## SUPPLEMENTARY MATERIALS FIGURES AND TABLES
















