# Supplementary material for: Analytic validation and real-time clinical application of an amplicon-based targeted gene panel for advanced cancer
Source: Oncotarget. 2017 Sep 1;8(44):75822–33. doi: 10.18632/oncotarget.20616 (PMC5652665; doi:10.18632/oncotarget.20616)
Supplement: Supplementary file 1 [file oncotarget-08-75822-s001.pdf]

Analytic validation and real-time clinical application of an amplicon-based targeted gene panel for advanced cancer

SUPPLEMENTARY MATERIALS

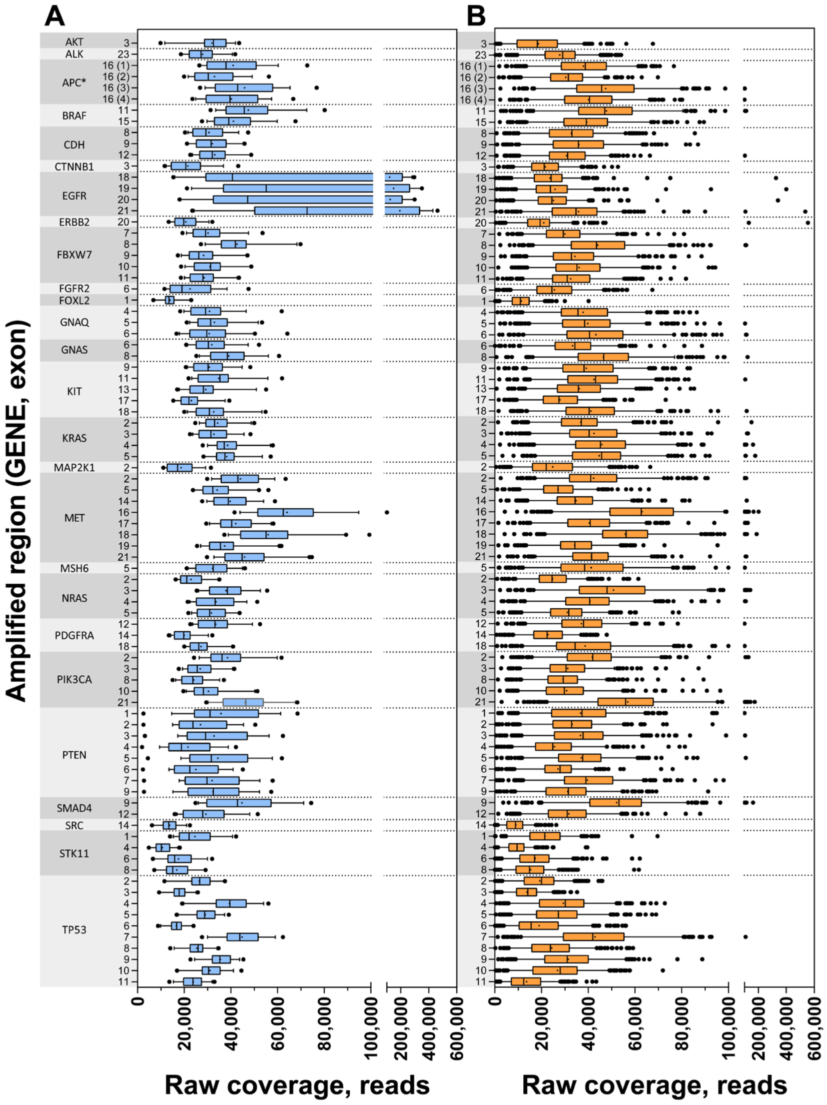

Figure S1. Raw coverage by amplicon. A) Forty independent validation samples and B) three hundred thirty-four independent patient samples (a tumor and germline sample for each of 167 patients) were sequenced using TST. The mean raw read depths for each of 85 regions across 26 genes are illustrated with the gene and exon covered listed on the Y axis. Box boundaries represent 25th and 75th percentiles, vertical lines within the boxes represent the median, a plus sign (+) within the box represents the mean, and whiskers represent 5th and 95th percentiles. Outliers beyond the 5th and 95th percentiles are illustrated as solid dots.

For Supplementary Tables see in Supplementary Files
